# Silver Dendritic Gels with Luminescence and Aggregation-Induced Emission Effect

**DOI:** 10.3390/gels10050291

**Published:** 2024-04-24

**Authors:** Verónica Iguarbe, Pilar Romero, Anabel Elduque, Raquel Giménez

**Affiliations:** 1 Instituto de Nanociencia y Materiales de Aragón (INMA), CSIC-Universidad de Zaragoza, 50009 Zaragoza, Spain; 2 Departamento de Química Orgánica, Facultad de Ciencias, Universidad de Zaragoza, 50009 Zaragoza, Spain; 3 Instituto de Síntesis Química y Catálisis Homogénea (ISQCH), CSIC-Universidad de Zaragoza, 50009 Zaragoza, Spain; 4 Departamento de Química Inorgánica, Facultad de Ciencias, Universidad de Zaragoza, 50009 Zaragoza, Spain

**Keywords:** metallogel, organogel, silver, dendrimers, luminescence, AIE

## Abstract

This work reports on a novel family of silver metallogels based on discrete coordination complexes. Structurally, they consist of dendrimers containing a trinuclear silver metallacycle at the core, with the general formula [M(μ-pz)]_3_, and poly(benzyl)ether branched structures with different numbers or terminal alkoxy chains at the periphery. These silver metallodendrimers are able to gel low-polarity solvents such as dodecane or cyclohexane, giving rise to luminescent organogels at room temperature with the property of aggregation-induced emission (AIE). This property means that in solution or the sol state, they are weak emitters, but in the gel state, luminescence is considerably increased. In this particular case, they exhibit blue luminescence. Two different dendritic scaffolds have been studied, finding significant differences in solubility, gel formation and dependence of luminescence on temperature. The results show that properly tailored silver gelators can show luminescence in the gel state.

## 1. Introduction

Among physical gels, molecular gels, those formed by the self-assembly of low-molar-mass gelators, contain advantageous characteristics for the development of smart soft materials [1,2,3]. Generally, these gelators encode information to give rise to one-dimensional fibrils by molecular self-assembly involving reversible interactions, such as van der Waals forces, hydrogen bonding, π-π interactions, solvophobic interactions and coordinated bonds. Then, the formed fibrils aggregate further into 3D networks, which are able to entrap and macroscopically immobilize a huge number of solvent molecules in the gel state. The number of solvent molecules can constitute even more than 99 wt% of the gelled material. Furthermore, the reversible nature of supramolecular interactions can be manipulated to develop molecular gels that switch reversibly between sol and gel states due to suitable external stimuli [4].

In addition, the use of gelators with luminescent properties can produce luminescent molecular gels, widening their prospects for applications in sensing, bioimaging, drug delivery and organic electronics and photonics [5,6,7]. Some gelators intrinsically exhibit the property of gelation-induced emission, a version of aggregation-induced emission (AIE), which means that in solution or the sol state, they are weak emitters, but in the gel state, luminescence is considerably increased due to favorable intermolecular interactions between the gelators and the restriction of molecular motion [8]. Those gelators emitting more strongly in the gelled state than in the sol state are especially important for developing highly photostable systems for biosensing [9] and testing the degree of aggregation by tracking the temporal dependences of gel formation [10], among other properties.

In addition to gels formed by low-molar-mass gelators, macromolecules of synthetic or natural origin can also give rise to gels due to self-assembly (physical polymeric gels). These polymeric gelators usually include multiple functionalities that favor entanglements to yield the gel network; however, they possess a less controllable structure. In between, and benefiting from the qualities of both low-molar-mass gels and polymeric gels, are those derived from dendritic structures. The regular and highly branched nature of dendrimers ensures the presence of multiple groups to establish supramolecular interactions while at the same time exhibiting reproducible self-assembly due to their well-defined molecular-size structure. Gelators based on different dendritic structures have been identified [11,12,13,14,15], with significant progress on poly(benzyl ether) dendrons and dendrimers [16]. Some examples report luminescent properties with an AIE effect [17,18,19,20,21,22,23,24].

The application of coordination chemistry to direct the self-assembly of molecules towards the formation of gels has opened a new field of research in the area of soft materials devoted to metallogels. In metallogels, the metal participates actively in the gel formation and introduces a distinctive behavior with respect to purely organic gels, such as differences in the gelation ability or morphology or the development of specific properties in the gel, including optical, catalytic, magnetic or redox properties. Either coordination polymers or discrete coordination complexes can produce metallogels, and they have a great diversity of structures and metallic ions, as evidenced in different review papers [25,26,27,28,29,30,31]. The formation of coordination polymers involves an elegant procedure to crosslink molecules and induce gel formation [32]. With respect to discrete coordination complexes, gold and platinum metallogels are remarkable due to their luminescent properties [33]. In contrast, silver metallogels made out of discrete coordination complexes are scarce [34,35,36], but they are reported to have interesting stimuli-responsive properties, such as ion responsiveness, or act as precursors for silver nanomaterials. Moreover, some examples of silver metallogels report luminescence quenching in the gel state [37].

**Figure 1:**
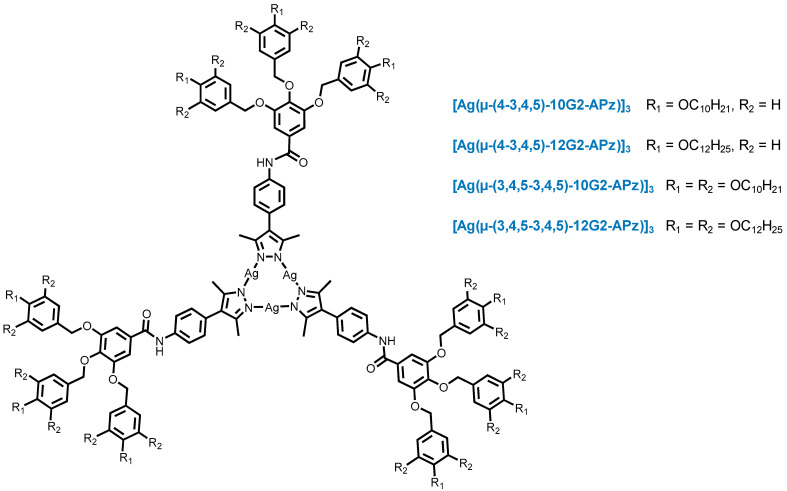
Silver metallodendrimers synthesized and studied in this work.

In this work, luminescent dendritic silver gelators are reported. They are based on novel discrete metal complexes with a cyclic trinuclear structure (CTC) comprising three metal atoms and three pyrazolate ligands with the general formula [M(μ-pz)]_3_. The CTC structure is known to exhibit luminescent properties for M = Cu and Au [38], and luminescent dendritic gels for M = Au have been reported [39]. In contrast, CTC structures with M = Ag are not luminescent at room temperature [40], and gels based on the silver metallacycle structure are unprecedented. In this work, silver metallogels that intrinsically show blue luminescence at room temperature with a significant AIE effect driven by gel formation are obtained. Different ligands derived from pyrazole dendrons have been used to prepare the metallodendrimers [41], which differ in the number and length of the terminal chains in the poly(benzyl)ether branched dendritic structure (Figure 1).

## 2. Results and Discussion

### 2.1. Synthesis and Characterization of the Silver Metallodendrimers

The synthesis of the silver trinuclear complexes was performed at room temperature using the previously reported pyrazole dendrons [17], with triethylamine as a base and AgPF_6_ as the silver source in equimolar amount with respect to the pyrazolate ligand. THF was chosen as the solvent for the reaction mixture due to the solubility of the pyrazole dendrons.

Metallodendrimers were characterized by several techniques in order to confirm the proposed chemical structure. The formation of the trinuclear species was verified by mass spectrometry. A molecular ion containing a silver or sodium atom was observed in the MALDI^+^ spectra. The analytical data were also in agreement with a 1:1 ligand-to-metal proportion.

The ^1^H NMR and ^13^C NMR spectra of the metallodendrimers show the same set of signals as their precursory dendrons due to their *C*_3_ symmetry. After complexation, a variation in the chemical shift occurs, being most significant for the signal corresponding to the methyl substituents at the pyrazole ring. Thus, the signal corresponding to these methyl protons appears at a lower field in the complexes, e.g., 2.36 ppm for **[Ag(µ-(4-3,4,5)-10G2-APz)]_3_** vs. 2.28 ppm in the corresponding 1*H*-pyrazole ligand. In the ^13^C-NMR spectra, the carbonyl corresponding to the amide group appears at 165–166 ppm.

**Figure 2:**
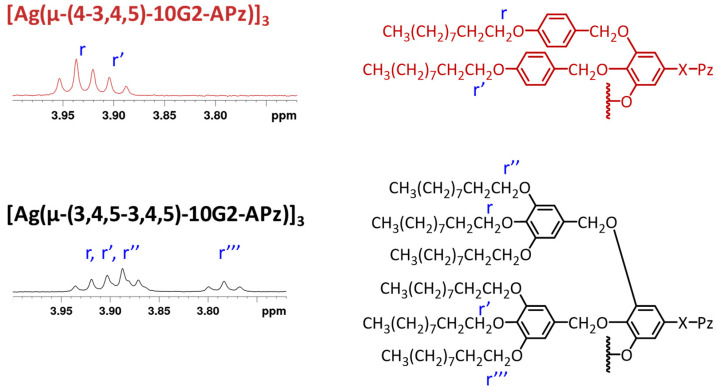
^1^H NMR methyleneoxy region and partial drawing of the dendritic structure to indicate the proton assignment.

It is interesting to note that by analyzing the ^1^H-NMR spectra at the region where the methylenoxy protons appear (3.7–4.0 ppm), it is possible to extract information about the structure of the metallodendrimers in solution. Compounds with a total of nine peripheral chains (three per dendron) show two superimposed triplets integrating 4:2, which correspond to the protons Hr and Hr′ (Figure 2, compound **[Ag(µ-(4-3,4,5)-10G2-APz)]_3_**). This pattern is typical for the benzyloxy groups, with a free rotation indicating that each dendron constituting the dendrimer is in a wedge-type conformation [42]. Metallodendrimers with a higher number of peripheral chains (nine per dendron) show three superimposed triplets (Hr, Hr′ and Hr″) and an isolated triplet at a higher field, corresponding to the more shielded Hr‴ protons (Figure 2, compound **[Ag(µ-(3,4,5-3,4,5)-10G2-APz)]_3_**). This is consistent with a non-flat conformation of the poly(benzyl)ether groups, and this is in agreement with the conformations found for the isolated precursory 1*H*-pyrazole dendrons [17].

In the FTIR spectra measured on KBr pellets, it is observed that, with the formation of metallodendrimers, the NH stretching band corresponding to the 1*H*-pyrazole ligand at 3182 cm^−1^ disappears (Figure S5 of the Supplementary Material). Typical amide group vibrations are observed in the associated region (N-H stretching at 3235 cm^−1^ and C=O stretching at 1644 cm^−1^), indicating the formation of hydrogen bonds in the prepared samples [17,43].

### 2.2. Study of Gels

#### 2.2.1. Gel Formation

To evaluate the potential of the synthesized silver metallodendrimers as gels, they were tested in different organic solvents in decreasing order of polarity (ethanol, ethyl acetate, dichloromethane, cyclohexane and dodecane) at a concentration of 5 wt% using the vial inversion test after cooling the mixture at room temperature. The results are shown in Table 1.

It is observed that silver metallodendrimers have distinct behavior depending on the number of terminal chains. Metallodendrimers with 4-3,4,5 substitution form gels in dodecane irrespective of the length of the terminal chains. In the rest of the solvents tested, the compounds were non-soluble. On the other hand, while the 3,4,5-3,4,5-substituted metallodendrimers were non-soluble in ethanol and ethyl acetate, they were soluble in dichloromethane and gave rise to a precipitate in more apolar solvents like cyclohexane and dodecane, except for **[Ag(µ-(3,4,5-3,4,5)-10G2-APz)]_3_**, which formed a gel in cyclohexane. Table 1 shows that the minimum gelation concentration was around 1 wt% for the 4-3,4,5 substitution and around 2 wt% for the 3,4,5-3,4,5 substitution.

From the obtained results, it is observed that there is a preference for low-polarity solvents, and the length of the terminal chain is less critical than the number of chains for gel formation. The different behaviors observed in changing the number of terminal chains come from the subtle interplay of intermolecular interactions in apolar solvents. As we have observed by NMR (see below), aggregation occurs through hydrogen bonding among the amide groups and π interactions at the inner part of the dendrimer, exposing the flexible chains at the periphery of the dendrimer to interactions with the apolar solvent. Fewer chains result in less solubility, and these dendrimers are only soluble in hot dodecane and form a gel upon cooling. In contrast, in cyclohexane, these dendrimers are non-soluble at any temperature. For dendrimers with a higher number of chains, the solubility increases, and they are soluble in dodecane and cyclohexane at high temperatures, but only **[Ag(µ-(3,4,5-3,4,5)-10G2-APz)]_3_** forms a gel upon cooling.

Table 2 shows the evolution of the temperature at which the gel transforms into a sol state (T_gel_) with concentration. In all cases, the temperature decreases as the concentration of the gel decreases, and it is higher for the metallodendrimers with a smaller number of peripheral chains. This indicates the formation of stronger aggregates.

The obtained metallogels are weaker than the organogels formed by the precursory pyrazole dendrons since the latter showed lower minimum gelation concentration in the supergel range (less than 1 wt%) [17]. This behavior can be attributed to the absence of hydrogen bonding intermolecular interactions, which 1*H*-pyrazoles can establish through their NH group with another 1*H*-pyrazole or with the amide groups. This absence is not compensated by the intermolecular interactions that the silver cyclic trinuclear structure can establish through π stacking, metallophilic interactions or hydrogen bonding interactions involving the amide groups.

**Figure 3:**
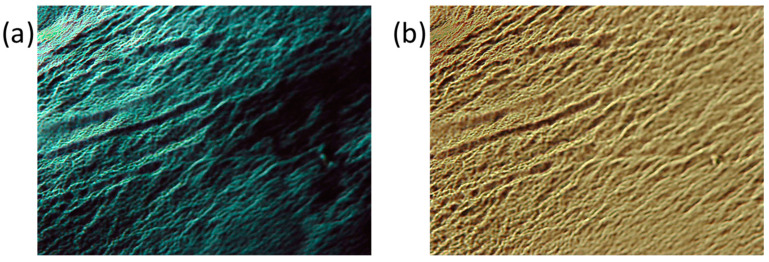
Microphotographs of a cyclohexane gel (2.5 wt%) of **[Ag(µ-(3,4,5-3,4,5)-10G2-APz)]_3_** observed (**a**) under crossed polarizer configuration and (**b**) under parallel polarizer configuration.

**Figure 4:**
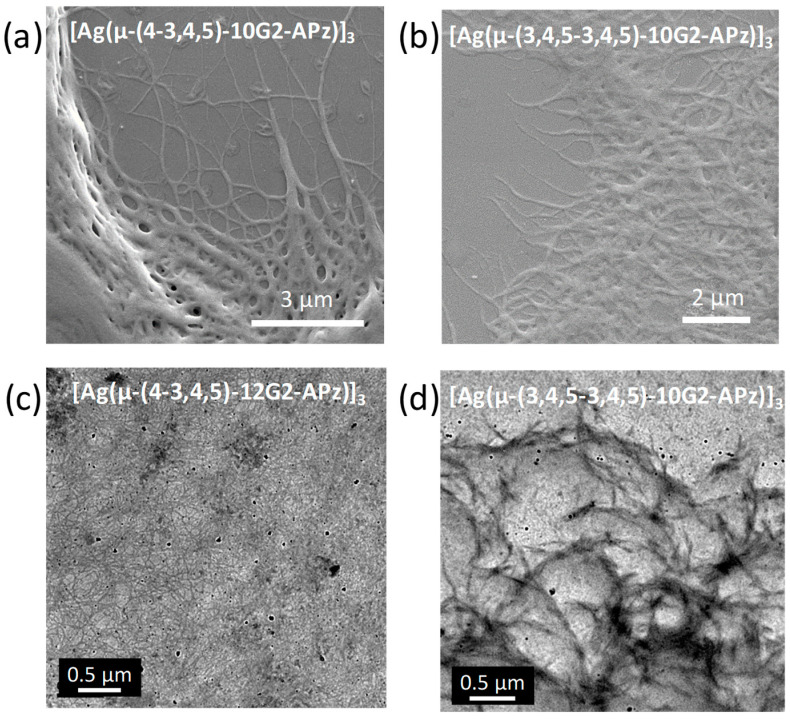
(**a**) SEM image of xerogel in dodecane (2 wt%), (**b**) SEM image of xerogel in cyclohexane (2.5 wt%), (**c**) TEM image of xerogel in dodecane (0.05 wt%), (**d**) TEM image of xerogel in cyclohexane (0.05 wt%).

Gels are opaque in aspect, and under the optical microscope with crossed polarizers, they appear as birefringent fibers distributed in an optically isotropic solvent (Figure 3a). Without polarized light, long fibers are also visualized (Figure 3b). SEM and TEM images gave rise to the visualization of fibrillar aggregates (Figure 4), although with less-defined morphology than those shown for the precursory 1*H*-pyrazole dendritic ligands [17]. Regarding the metallogels, SEM analysis indicates that xerogels have a high tendency to form dense networks constituted by agglomerated fibers. These fibers were only clearly observed at the edges of the sample preparation. Based on this, diluted samples were prepared in order to properly observe the fibers using the TEM technique.

#### 2.2.2. NMR Study

**Figure 5:**
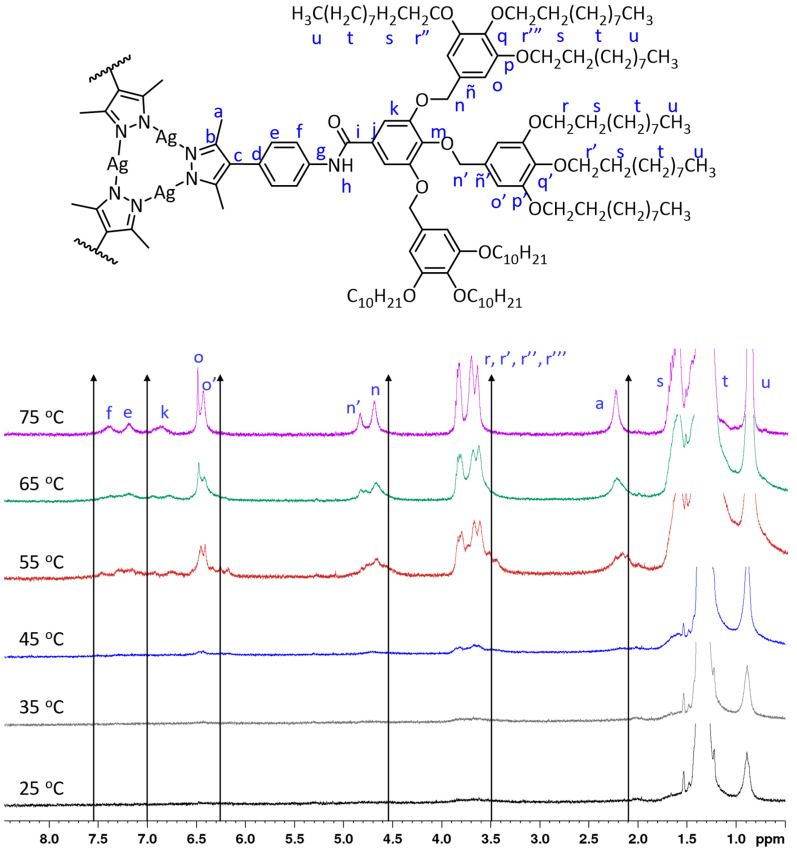
^1^H NMR spectra (400 MHz) obtained by heating a **[Ag(μ-(3,4,5-3,4,5)-10G2-APz)]_3_** gel in C_6_D_12_ (3 wt%) at different temperatures and proton signal assignment.

In order to investigate the supramolecular interactions involved in gel formation, the ^1^H-NMR spectra of a gel formed in deuterated cyclohexane (C_6_D_12_) were recorded for **[Ag(µ-(3,4,5-3,4,5)-10G2-APz)]**_3_ at different temperatures (Figure 5). At room temperature, due to the low mobility of the molecules in the gel state, signals were hardly observed in the spectra. As the temperature gradually increased, upon exceeding the T_gel_ (35–40 °C), the signals corresponding to the aromatic protons, the methylene groups and the methyl groups at the 3 and 5 positions of the pyrazole ring began to be observed since the material was in a partially gel state and the number of isolated molecules in solution increased due to the breakdown of aggregates. Specifically, the signal H_o_ was well defined, indicating that the dendritic part became less rigid. Moreover, the signals corresponding to the aromatic protons, the methylene groups adjacent to the benzene rings and the methyl groups of the pyrazole ring were observed to move to higher ppm with increasing temperature. This partial deshielding could be indicative of the weakening of π stacking interactions upon gel–sol transformation.

For gels formed by the precursory 1*H*-pyrazole dendrons, the greatest variation with increasing temperature was found for the proton of the amide group (H_h_), which was indicative of the breakage of hydrogen bonds by increasing temperature [17]. For this metallodendrimer, it could not be observed if there was any variation in the H_h_ signal because the aromatic region, or where it was expected to be, was not resolved at high temperatures. This indicates that, although metallogels are weaker than 1*H*-pyrazole dendron gels, they form different and stronger aggregates in the sol state.

**Figure 6:**
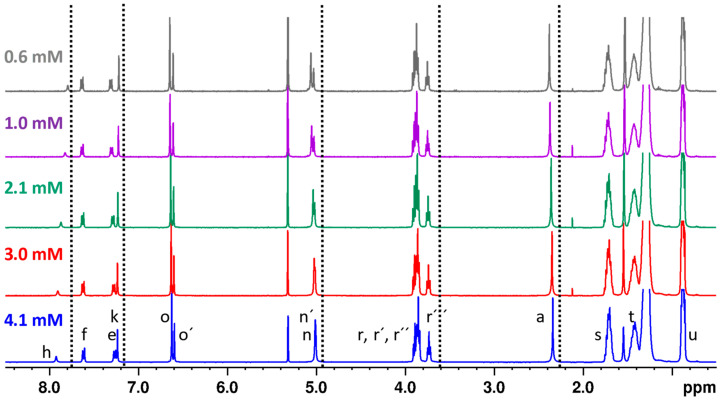
^1^H-NMR spectra at room temperature for the compound **[Ag(µ-(3,4,5-3,4,5)-10G2-APz)]_3_** in CD_2_Cl_2_ at different concentrations. Refer to Figure 5 for proton assignment.

In order to elucidate the existence of hydrogen bonding interactions through amide groups and test the effect on the H_h_ signal, we obtained ^1^H NMR spectra in a solvent in which the molecule shows higher solubility; in this case, we studied CD_2_Cl_2_ solutions at different concentrations at room temperature (Figure 6). In general, a slight dependence of some of the signals with increasing concentration was observed, indicating a low tendency to aggregation in this solvent. In the case of the NH protons of the amide bond (H_h_) and the protons of the benzene ring (H_k_), a slight shift to higher ppm was observed with increasing concentration, that is, in the opposite direction to the rest of the protons. This shift indicates that hydrogen bonds are formed as the concentration increases. In the methyleneoxy protons (Hr‴), as well as the methyl substituents of the pyrazole ring (H_a_) and the benzylic CH_2_ (H_n_ and H_n_′), a slight shift to lower ppm was observed with increasing concentration. This shielding is due to the stacking of the rings through π-π interactions. The variation in the chemical shift vs. concentration allowed the quantitative determination of the association constant (K = 143 ±19 M^−1^) (see Figure S6 of Supplementary Materials). In conclusion, although low aggregation takes place in this solvent, it can be confirmed that hydrogen bonding through amide groups, together with π stacking interactions, plays a role in the molecular aggregation and formation of metallogels.

#### 2.2.3. Luminescence and AIE Properties

**Figure 7:**
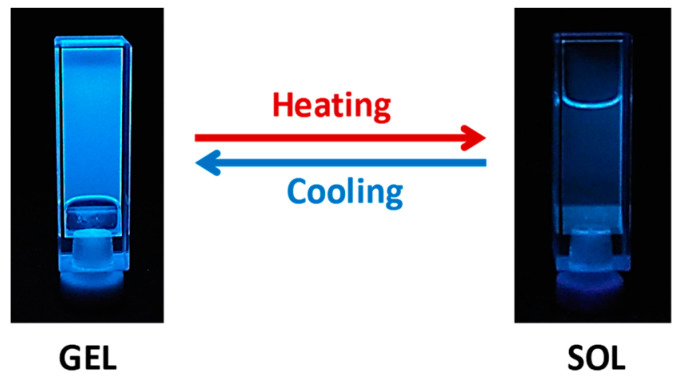
Gel and sol states for **[Ag(µ-(3,4,5-3,4,5)-10G2-APz)]_3_** (3 wt% in cyclohexane) under irradiation with a UV lamp.

As commented in the introduction, silver metallogels are not usually known for their luminescent properties, and silver-derived CTC structures were not reported to form gels. With these silver gelators, all gels display blue luminescence at room temperature (Figure 7). The emission band was similar to that found for gels based on the 1*H*-pyrazole precursor dendrons, indicating the essential influence of the pyrazole ligand structure [17].

**Figure 8:**
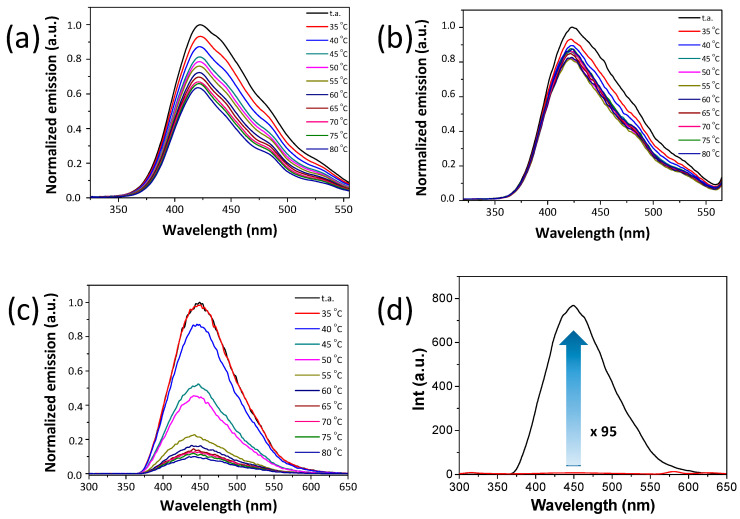
Emission spectra at different temperatures for (**a**) **[Ag(µ-(4-3,4,5)-10G2-APz)]_3_** (dodecane, 2 wt%), (**b**) **[Ag(µ-(4-3,4,5)-12G2-APz)]_3_** (dodecane, 1.7 wt%), (**c**) **[Ag(µ-(3,4,5-3,4,5)-10G2-APz)]_3_** (cyclohexane, 3 wt%). (**d**) Emission spectra at room temperature of a gel (cyclohexane, 3 wt%) (black trace) and a solution (THF, 3 wt%) (red trace) and the calculated AIE effect for **[Ag(µ-(3,4,5-3,4,5)-10G2-APz)]_3_**.

On heating the gels to acquire the sol state, a decrease in the emission was observed. The sol state was barely luminescent (Figure 7). Table 3 shows the emission data for the synthesized metallodendrimers in the gel (room temperature) and sol (80 °C) states. All gels showed a broad emission band around 423–450 nm that suffered from a slight blue shift on heating to the sol state. Concretely, luminescence intensity was maximum at room temperature in the gel state and decreased significantly when surpassing the T_gel_ (Figure 8a–c). The decrease was more abrupt in the case of the metallogel of compound **[Ag(µ-(3,4,5-3,4,5)-10G2-APz)]_3_**, which points to an important influence of the dendritic structure, as this structure seems to have more bulkiness in solution (see above). The luminescence was recovered by reforming the gel on cooling.

In order to study the effect of aggregation on the luminescent properties, we measured the emission spectra of the synthesized metallodendrimers in diluted solutions. They showed a band with a maximum between 345 and 356 nm in THF at a concentration of 10^−6^ M (Table 3). That is, in diluted solution, these molecules show the emission band in the UV region, and show the emission band in the visible region in the aggregated states.

In addition, in order to demonstrate that the emission increase when cooling the sol to the gel state solely occurs due to gel formation and not due to the favorable decreasing temperature effect, the luminescence intensity of the gels at room temperature was compared with that of a solution of THF at room temperature of the same concentration (Table 3, Figure 8d). The emission from the concentrated THF solution was hardly detectable. The emission increase can be calculated to be up to 95 times in the gel state. In general, an increase in emission of two orders of magnitude is observed for these silver metallogels. This means that the metallodendrimers are able to enhance the luminescence through gel formation.

## 3. Conclusions

This work shows that it is possible to obtain silver metallogels with luminescence and AIE effect. The combination of the silver CTC structure at the core and a dendritic periphery based on a polyalkoxylated poly(benzyl)ether scaffold gives rise to the formation of organogels in dodecane or cyclohexane at relatively low concentrations. ^1^H NMR studies evidence the existence of π stacking and hydrogen bonds throughout the amide groups. Nevertheless, the minimum gelation concentrations are higher than those found for the precursory 1*H*-pyrazole dendrons, which are able to interact via additional hydrogen bonds through the NH pyrazole groups.

Two different dendritic scaffolds have been studied, finding significant differences in solubility, gel formation and dependence of luminescence on temperature. The most abrupt decrease in luminescence upon heating to the sol state is found for the gel obtained for the **[Ag(µ-(3,4,5-3,4,5)-10G2-APz)]_3_** metallodendrimer. In addition, it shows the highest AIE effect through comparison of the gel with a THF solution at the same concentration and at the same temperature, evidencing the importance of the dendritic substitution on the metallogel properties.

## 4. Materials and Methods

### 4.1. Experimental Techniques for the Synthesis and Characterization of the Novel Compounds

Pyrazole dendrons used as ligands for the preparation of silver trinuclear complexes were synthesized as previously reported [17]. Triethylamine and AgPF_6_ were purchased from Aldrich and used without further purification. Solvents (THF, methanol and dichloromethane) were purchased from Fisher Scientific. THF and dichloromethane were dried using a PureSolv solvent purification system. Reactions were performed under argon using Schlenk techniques, and the glassware was oven-dried (110 °C) prior to use.

^1^H-NMR and ^13^C-NMR spectra were acquired using a Bruker AV400 spectrometer (Bruker Corp, Billerica, MA, USA). Chemical shifts are given in ppm relative to tetramethylsilane (TMS), and the solvent residual peak (CD_2_Cl_2_ or C_2_D_2_Cl_4_) was used as an internal standard. Metallodendrimers belonging to the 4-3,4,5 series are not soluble at high concentrations in CD_2_Cl_2_, and in some cases, the experiments were carried out in C_2_D_2_Cl_4_ at high temperatures, as indicated in each particular case. Mass spectra were obtained using a MICROFLEX Bruker spectrometer by MALDI in the positive mode using ((2E)-2-Methyl-3-[4-(2-methyl-2-propanyl)phenyl]-2-propen-1-ylidene)malononitrile (DCTB) as the matrix. Elemental analyses were performed using a Perkin–Elmer 2400 series II microanalyzer (PerkinElmer Inc., Shelton, CT, USA). Fluorescence spectra were recorded with a Perkin–Elmer LS50B system (PerkinElmer Inc., Shelton, CT, USA) using 1 cm quartz cells and chromatographic-grade purity solvents. Polarized optical microscopy was performed using an Olympus BX51 microscope equipped with an Olympus DP22 camera (Olympus Corporation, Hachioji-shi, Tokyo) using a 10× objective lens. Scanning electron microscopy (SEM) studies were performed with an Inspect F50 instrument (FEI Company, Eindhoven, The Netherlands). Samples were prepared by casting the gel onto glass and drying under a vacuum to obtain the xerogel. Then, the xerogels were covered with a Pd layer of 14 nm thickness. Transmission electron microscopy (TEM) measurements were taken on a JEOL-2000-FXIII operating (JEOL Ltd., Akishima-Shi, Tokyo) at 200 kV. A drop of the sample was deposited onto holey carbon copper grids, and the solvent was evaporated. Then, the samples were stained with uranyl acetate (1 wt% in water) and dried at room temperature.

### 4.2. Synthesis and Characterization of the Silver Dendritic Complexes

All silver complexes (Figure 1) were synthesized following a general procedure as follows. A Schlenk flask was charged with the corresponding dendritic pyrazole ligand (0.11 mmol), AgPF_6_ (0.11 mmol) and dry THF (10 mL). The mixture was degassed and stirred for 5 min under an argon atmosphere. Then, triethylamine (0.13 mmol) was added, and the mixture was stirred at room temperature in darkness for 15 h.

Metallodendrimers with 4-3,4,5 substitution were isolated by evaporation of the reaction mixture to a volume of 1 mL, and precipitation by addition of 10 mL of methanol. The obtained white solid was filtered and dried under vacuum.

Metallodendrimers with 3,4,5-3,4,5 substitution were isolated using a different procedure. The reaction mixture was evaporated to dryness, then 10 mL of dichloromethane was added and the mixture was filtered through a pad of celite. Then, the filtrate was evaporated in the vacuum line up to a volume of 1 mL, and 10 mL of methanol was added. The obtained white solid was filtered and dried under vacuum.

**[Ag(µ-(4-3,4,5)-10G2-APz)]_3_**: White solid. Yield: 87%. ^1^H-NMR (400 MHz, C_2_D_2_Cl_4_, 60 °C) δ (ppm): 0.90–0.94 (m, 27H, C*H*_3_(CH_2_)_7_), 1.32–1.52 (m, 126H, CH_3_(C*H*_2_)_7_), 1.80–1.84 (m, 18H, C*H*_2_CH_2_OAr), 2.37 (s, 18H, C*H*_3_Pz), 3.95–4.02 (m, 18H, CH_2_C*H*_2_OAr), 5.04 (s, 6H, ArC*H*_2_OAr), 5.10 (s, 12H, ArC*H*_2_OAr), 6.81–6.84 (m, 6H, AA’XX’), 6.94–6.96 (m, 12H, AA’XX’), 7.19 (s, 6H, Ar-*H*), 7.30–7.33 (m, 6H, AA’XX’), 7.34–7.38 (m, 18H, Ar-*H*), 7.64–7.67 (m, 6H, AA’XX’), 7.71 (s, 3H, ArCON*H*_2_). ^13^C-NMR: (100 MHz, C_2_D_2_Cl_4_, 60 °C) δ (ppm): 13.7, 14.2, 22.7–31.9, 68.3, 68.4, 71.7, 75.0, 107.6, 114.5, 114.9, 117.3, 120.7, 128.6, 129.4, 129.6, 129.8, 130.3, 130.6, 131.2, 135.6, 142.2, 147.1, 153.1, 159.2, 159.3, 165.5. MS (MALDI^+^, DCTB) *m*/*z*: 3663.3 [M + Ag]^+^. Elemental analysis: calcd for (%) C_207_H_282_Ag_3_N_9_O_21_: C 69.91, H 7.99, N 3.54; found: C 70.16, H 8.21, N 3.49.
**[Ag(µ-(4-3,4,5)-12G2-APz)]_3_**: White solid. Yield: 89%. ^1^H-NMR (400 MHz, C_2_D_2_Cl_4_, 95 °C) δ (ppm): 0.94–0.97 (m, 27H, C*H*_3_(CH_2_)_9_), 1.35–1.54 (m, 162H, CH_3_(C*H*_2_)_9_), 1.80–1.88 (m, 18H, C*H*_2_CH_2_OAr), 2.37 (s, 18H, C*H*_3_Pz), 3.99–4.06 (m, 18H, CH_2_C*H*_2_OAr), 5.10 (s, 6H, ArC*H*_2_OAr), 5.14 (s, 12H, ArC*H*_2_OAr), 6.85–6.87 (m, 6H, AA’XX’), 6.96–6.98 (m, 12H, AA’XX’), 7.22 (s, 6H, Ar-*H*), 7.33–7.40 (m, 24H, Ar-*H*), 7.61 (s, 3H, ArCON*H*_2_), 7.66–7.68 (m, 6H, AA’XX’). ^13^C-NMR: (100 MHz, C_2_D_2_Cl_4_, 95 °C) δ (ppm): 13.5, 14.0, 22.5–31.7, 68.1, 68.2, 71.5, 74.8, 107.4, 114.3, 114.6, 117.0, 120.5, 128.4, 129.1, 129.4, 129.6, 130.0, 130.7, 135.4, 142.0, 145.0, 152.9, 159.0, 159.1, 165.3. MS (MALDI^+^, DCTB) *m*/*z*: 3920.3 [M + Ag]^+^. Elemental analysis: calcd for (%) C_225_H_318_Ag_3_N_9_O_21_: C 70.96, H 8.42, N 3.31; found: C 70.68, H 8.75, N 3.52.
**[Ag(µ-(3,4,5-3,4,5)-10G2-APz)]_3_**: White solid. Yield: 79%. ^1^H-NMR (400 MHz, CD_2_Cl_2_) δ (ppm): 0.86–0.90 (m, 81H, C*H*_3_(CH_2_)_7_), 1.27–1.47 (m, 378H, CH_3_(C*H*_2_)_7_), 1.66–1.76 (m, 54H, C*H*_2_CH_2_OAr), 2.36 (s, 18H, C*H*_3_Pz), 3.74 (t, 12H, *J* = 6.6 Hz, CH_2_C*H*_2_OAr), 3.84–3.91 (m, 42H, CH_2_C*H*_2_OAr), 5.01 (s, 6H, ArC*H*_2_OAr), 5.03 (s, 12H, ArC*H*_2_OAr), 6.60 (s, 6H, Ar-*H*), 6.63 (s, 12H, Ar-*H*), 7.23 (s, 6H, Ar-*H*), 7.27–7.30 (m, 6H, AA’XX’), 7.61–7.64 (m, 6H, AA’XX’), 7.88 (s, 3H, ArCON*H*_2_). ^13^C-NMR (100 MHz, CD_2_Cl_2_) δ (ppm): 14.3, 14.5, 23.3–32.5, 69.4, 69.5, 72.4, 73.8, 73.9, 75.7, 106.2, 106.4, 107.8, 118.4, 121.0, 130.1, 131.1, 132.1, 132.3, 133.1, 136.2, 138.2, 138.3, 142.0, 147.7, 153.5, 153.6, 153.9, 165.7. MS (MALDI^+^, DCTB) *m*/*z*: 6392.1 [M + Na]^+^, 6476.2 [M + Ag]^+^. Elemental analysis: calcd for (%) C_387_H_642_Ag_3_N_9_O_39_: C 72.98, H 10.16, N 1.96; found: C 72.61, H 10.23, N 2.12.
**[Ag(µ-(3,4,5-3,4,5)-12G2-APz)]_3_**: White solid. Yield: 93%. ^1^H-NMR (400 MHz, CD_2_Cl_2_) δ (ppm): 0.87–0.90 (m, 81H, C*H*_3_(CH_2_)_9_), 1.26–1.47 (m, 486H, CH_3_(C*H*_2_)_9_), 1.70–1.75 (m, 54H, C*H*_2_CH_2_OAr), 2.36 (s, 18H, C*H*_3_Pz), 3.73 (t, 12H, *J* = 6.6 Hz, CH_2_C*H*_2_OAr), 3.84–3.91 (m, 42H, CH_2_C*H*_2_OAr), 5.00 (s, 18H, ArC*H*_2_OAr), 6.60 (s, 6H, Ar-*H*), 6.62 (s, 12H, Ar-*H*), 7.24–7.27 (m, 12H, Ar-*H*), 7.61-7.63 (m, 6H, AA’XX’), 7.94 (s, 3H, ArCON*H*_2_). ^13^C-NMR (100 MHz, CD_2_Cl_2_) δ (ppm): 14.3, 14.5, 23.3-32.5, 69.4, 69.6, 72.3, 73.8, 73.9, 75.7, 106.2, 106.4, 107.8, 116.6, 121.1, 130.0, 130.1, 131.1, 132.3, 133.1, 136.2, 138.2, 138.3, 142.0, 149.3, 153.5, 153.6, 153.9, 166.1. MS (MALDI^+^, DCTB) *m*/*z*: 7146.2 [M + Na]^+^. Elemental analysis: calcd for (%) C_441_H_750_Ag_3_N_9_O_39_: C 74.33, H 10.61, N 1.77; found: C 74.61, H 10.69, N 1.70.

## Figures and Tables

**Table 1:** Gel formation study for the metallodendrimers at a concentration of 5 wt% ^a^.

Compound	Ethanol	Ethyl Acetate	Dichloromethane	Cyclohexane	Dodecane
**[Ag(µ-(4-3,4,5)-10G2-APz)]_3_**	I	I	I	I	G [1.3]
**[Ag(µ-(4-3,4,5)-12G2-APz)]_3_**	I	I	I	I	G [1.1]
**[Ag(µ-(3,4,5-3,4,5)-10G2-APz)]_3_**	I	I	S	G [2.2]	P
**[Ag(µ-(3,4,5-3,4,5)-12G2-APz)]_3_**	I	I	S	P	P

^a^ I: non-soluble, G: gel, S: soluble, P: precipitate. In brackets is shown the minimum gelation concentration in wt%.

**Table 2:** Temperatures (°C) for the gel-to-sol transition (T_gel_) at different concentrations.

Compound	5 wt%	4 wt%	3 wt%	2 wt%
**[Ag(µ-(4-3,4,5)-10G2-APz)]_3_**	81 ^a^	75 ^a^	66 ^a^	56 ^a^
**[Ag(µ-(4-3,4,5)-12G2-APz)]_3_**	86 ^a^	78 ^a^	70 ^a^	64 ^a^
**[Ag(µ-(3,4,5-3,4,5)-10G2-APz)]_3_**	54 ^b^	41 ^b^	35 ^b^	-

^a^ Dodecane. ^b^ Cyclohexane.

**Table 3:** Photoluminescence data of the metallodendrimers.

Compound	λ_gel_ ^a^ (nm)	λ_sol_ ^b^ (nm)	λ_THFdil_^a,c^ (nm)	λ_THFconc_^a,d^ (nm)	AIE
**[Ag(µ-(4-3,4,5)-10G2-APz)]_3_**	423 ^e,g^	418 ^e,g^	356	440 ^g^	61
**[Ag(µ-(4-3,4,5)-12G2-APz)]_3_**	425 ^e,g^	419 ^e,g^	346	445 ^g^	58
**[Ag(µ-(3,4,5-3,4,5)-10G2-APz)]_3_**	449 ^f,h^	439 ^f,h^	355	453 ^h^	95

^a^ Room temperature, ^b^ 80 °C, ^c^ 10^−6^ M THF solution, ^d^ concentrated THF solution, ^e^ dodecane, ^f^ cyclohexane. ^g^ 2 wt%. ^h^ 3 wt%.

## Data Availability

Data are included in the article and the Supplementary Materials.

## Supplementary Materials

The following supporting information can be downloaded at: https://www.mdpi.com/article/10.3390/gels10050291/s1, Figure S1: ^1^H-NMR (400 MHz, C_2_D_2_Cl_4_, 60 °C) and ^13^C-NMR (100 MHz, C_2_D_2_Cl_4_, 60 °C) spectra of **[Ag(µ-(4-3,4,5)-10G2-APz)]_3_**. Figure S2: ^1^H-NMR (400 MHz, C_2_D_2_Cl_4_, 60 °C) and ^13^C-NMR (100 MHz, C_2_D_2_Cl_4_, 60 °C) spectra of **[Ag(µ-(4-3,4,5)-12G2-APz)]_3_**. Figure S3: ^1^H-NMR (400 MHz, CD_2_Cl_2_, 25 °C) and ^13^C-NMR (100 MHz, CD_2_Cl_2_, 25 °C) spectra of **[Ag(µ-(3,4,5-3,4,5)-10G2-APz)]_3_**. Figure S4: ^1^H-NMR (400 MHz, CD_2_Cl_2_, 25 °C) and ^13^C-NMR (100 MHz, CD_2_Cl_2_, 25 °C) spectra of **[Ag(µ-(3,4,5-3,4,5)-12G2-APz)]_3_**. Figure S5: FTIR spectra in KBr pellets for (a) ligand precursor 3,4,5-3,4,5-10G2-APzH and (b) their corresponding metallodendrimers **[Ag(µ-(3,4,5-3,4,5)-10G2-APz)]_3_**. Figure S6: Concentration dependence of NMR chemical shifts of the H_h_, H_k_ and H_a_ signals for **[Ag(µ-(3,4,5-3,4,5)-10G2-APz)]_3_** in CD_2_Cl_2_. Refer to Figure 5 or Figure S3 for proton assignment.
