# Supplementary material for: Silver Dendritic Gels with Luminescence and Aggregation-Induced Emission Effect
Source: Gels. 2024 Apr 24;10(5):291. doi: 10.3390/gels10050291 (PMC11120864; doi:10.3390/gels10050291)
Supplement: Supplementary file 1 [file gels-10-00291-s001.zip › gels-2952908-supplementary.pdf]

Supplementary Materials:

Silver Dendritic Gels with Luminescence and AIE effect

Verónica Iguarbe [1], Pilar Romero [1], Anabel Elduque [2,*] and Raquel Giménez [1,*]

[1] Instituto de Nanociencia y Materiales de Aragón (INMA), CSIC-Universidad de Zaragoza, 50009 Zaragoza, Spain.
[2] Departamento de Química Orgánica, Facultad de Ciencias, Universidad de Zaragoza, 50009 Zaragoza, Spain.
[3] Instituto de Síntesis Química y Catálisis Homogénea (ISQCH), CSIC-Universidad de Zaragoza, 50009 Zaragoza, Spain
[4] Departamento de Química Inorgánica, Facultad de Ciencias, Universidad de Zaragoza, 50009 Zaragoza, Spain.
* Correspondence: anaelduq@unizar.es (A.E.); rgimenez@unizar.es (R.G.)

Index Page

Index	Page
**Figure S1.**	**2**
**Figure S2**	**3**
**Figure S3**	**4**
**Figure S4**	**5**
**Figure S5**	**6**
**Calculation of the association constant**	**7**
**Figure S6**	**7**

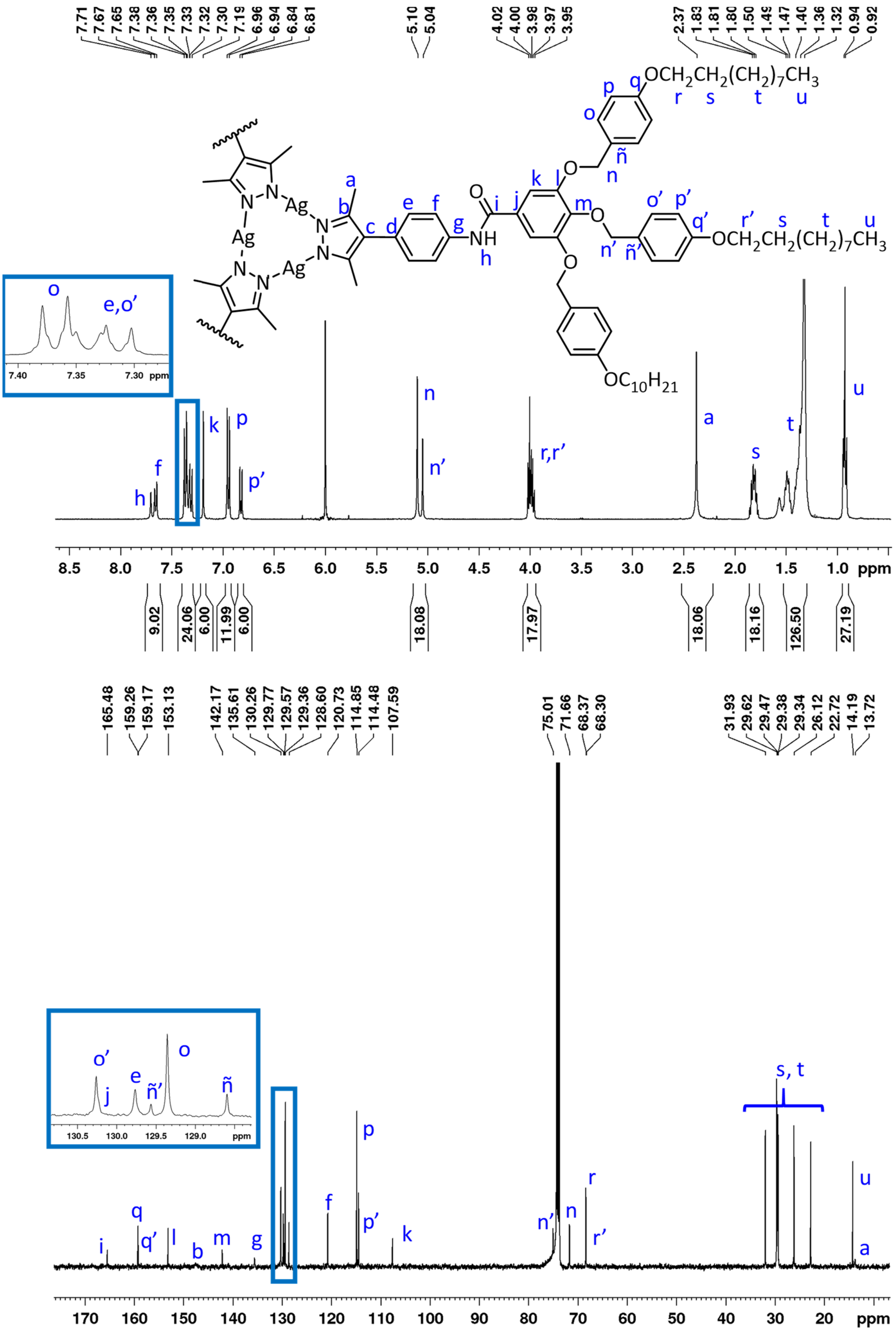

Figure S1. ^{1}H-NMR (400 MHz, $C_2D_2Cl_4$, 60 °C) and ^{13}C-NMR (100 MHz, $C_2D_2Cl_4$, 60 °C) spectra of **[Ag(μ-(4-3,4,5)-10G2-APz)]$_3$.**

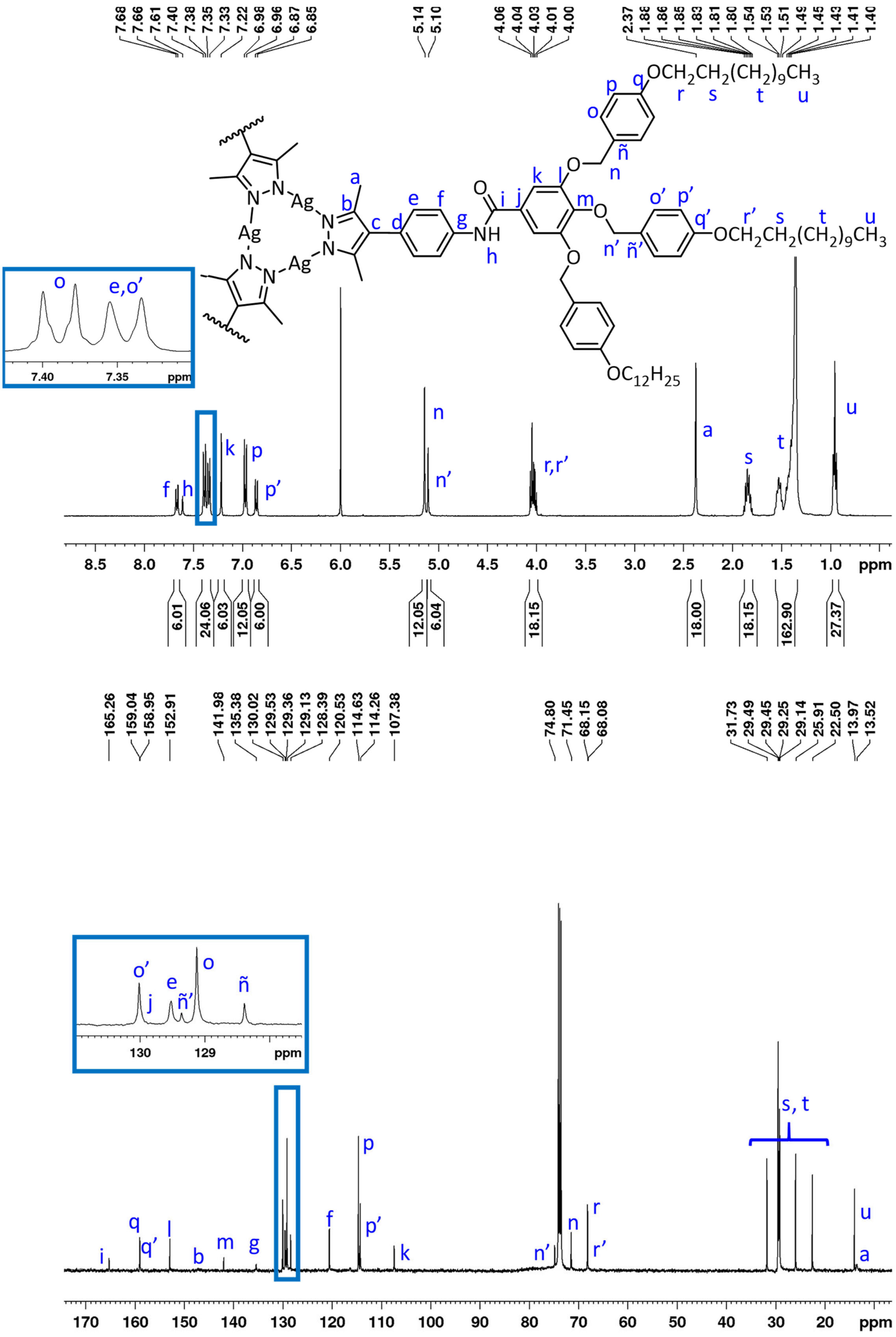

Figure S2. ^{1}H-NMR (400 MHz, $C_2D_2Cl_4$, 60 °C) and ^{13}C-NMR (100 MHz, $C_2D_2Cl_4$, 60 °C) spectra of **[Ag(μ-(4-3,4,5)-12G2-APz)]**$_3$.

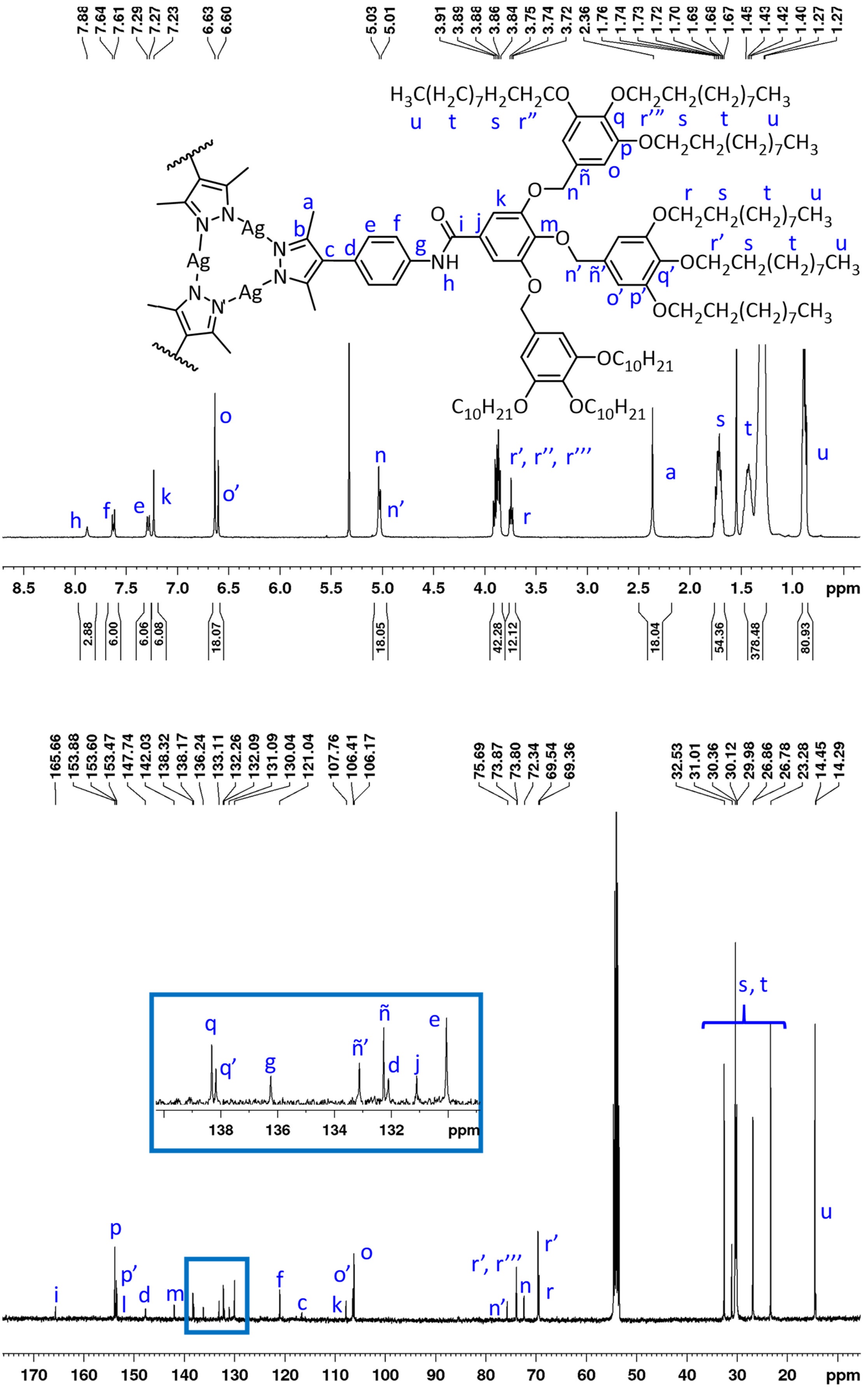

Figure S3. ^{1}H-NMR (400 MHz, CD_2Cl_2, 25 °C) and ^{13}C-NMR (100 MHz, CD_2Cl_2, 25 °C) spectra of **[Ag(μ-(3,4,5-3,4,5)-10G2-APz)]$_3$.**

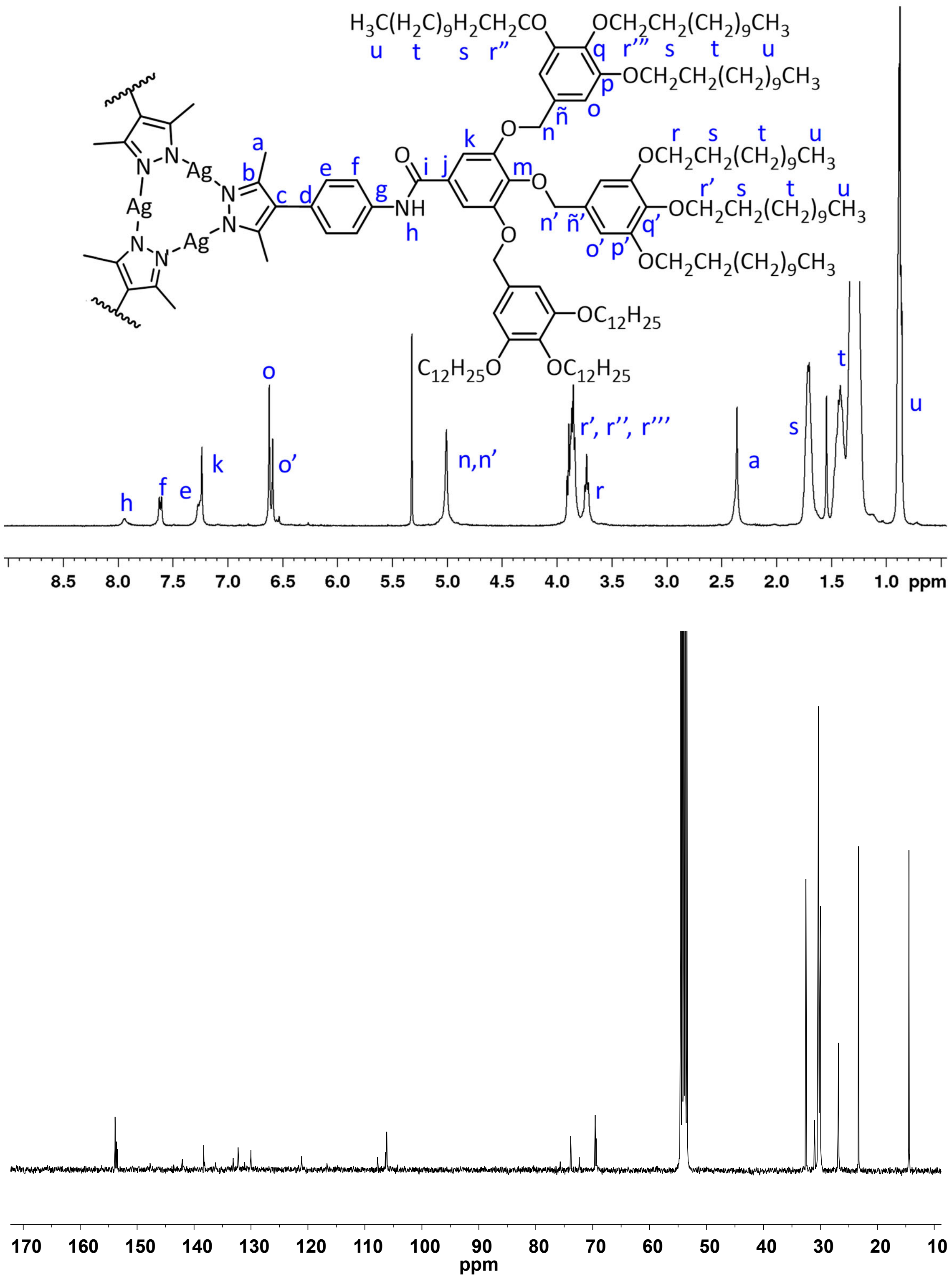

Figure S4. ^{1}H-NMR (400 MHz, CD_2Cl_2, 25 ºC) and ^{13}C-NMR (100 MHz, CD_2Cl_2, 25 ºC) spectra of **[Ag(μ-(3,4,5-3,4,5)-12G2-APz)]**$_3$.

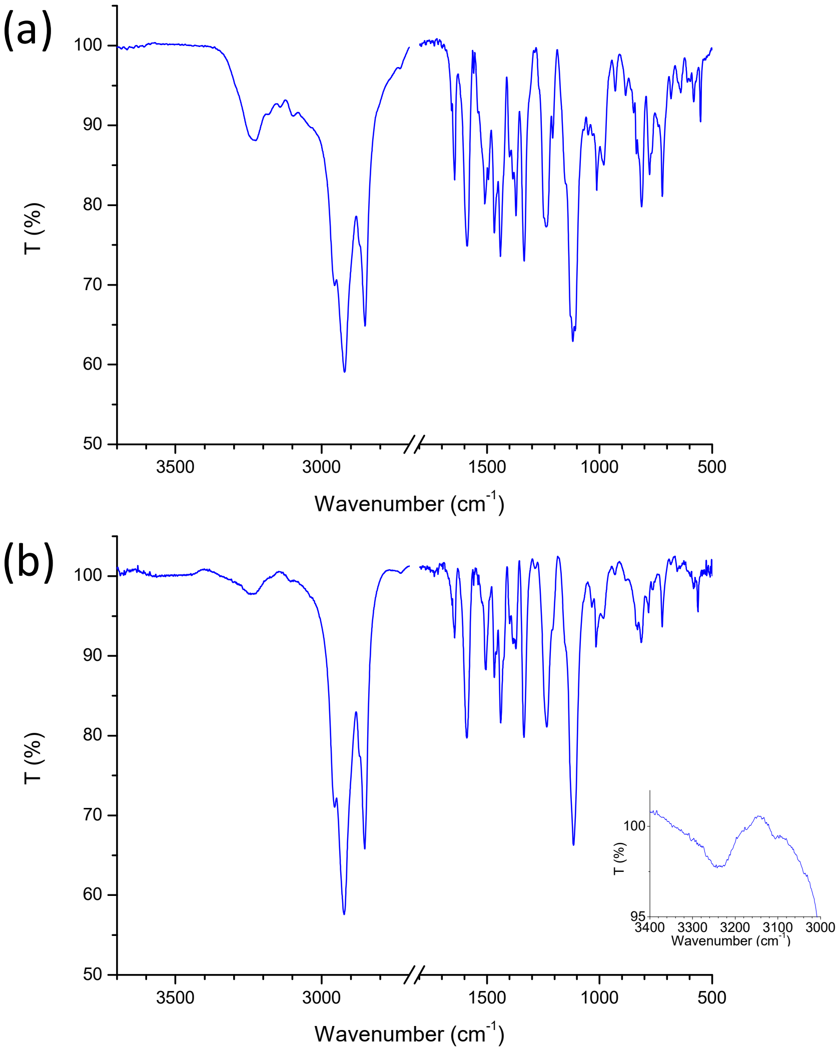

Figure S5. FTIR spectra in KBr pellets for (a) ligand precursor **3,4,5-3,4,5-10G2-APzH** and (b) their corresponding metallodendrimer **[Ag(μ-(3,4,5-3,4,5)-10G2-APz)]**$_3$.

Calculation of the association constant

Curve fitting was performed using nonlinear regression analysis (OriginPro 2020b, version 9.7 (Academic) by OriginLab Corporation) assuming the isodesmic model according to the equation:

$$\delta_{obs} = \delta_m + (\delta_{ag} - \delta_m)\left(1 + \frac{(1 - \sqrt{4KC_T + 1})}{2KC_T}\right)$$

in which K is the association constant, C_T is the total concentration, δ_{obs} is the measured chemical shift at concentration C_T, δ_m and δ_{ag} are limiting chemical shifts of monomer and the aggregate, respectively. Values and curve fitting obtained for three signals were as follows:

Signal	NH (H_h)	H_k	H_a
K (M^{-1})	142.69861±19.6257	142.69861±19.6257	142.69861±19.6257
R-square	0.99368	0.99186	0.9963

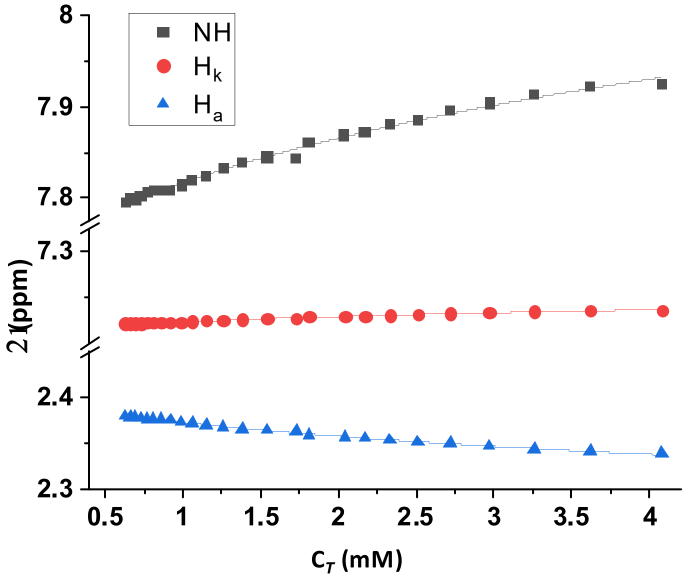

Figure S6. Concentration dependence of NMR chemical shifts of the H_h, H_k and H_a signals for **[Ag(μ-(3,4,5-3,4,5)-10G2-APz)]$_3$** in CD_2Cl_2. Refer to Figure 5 or Figure S3 for proton assignment.
